# Access to Information on Needed Services Mediates the Association Between Income and Self-Rated Health

**DOI:** 10.1093/geroni/igaa057.349

**Published:** 2020-12-16

**Authors:** Sara Moss, Katie White, Holly Dabelko-Schoeny

**Affiliations:** 1 Ohio State University, Columbus, Ohio, United States; 2 Age Friendly Columbus, Columbus, Ohio, United States

## Abstract

Prior research has documented that individuals with fewer financial resources report lower self-rated health. The relationship between financial status and health is important to understand among older adults, for whom income is often fixed, and who face unique health stressors. In this study, we examined whether physical activity, connection to community, and access to information, variables which have demonstrated associations with financial resources and health in previous studies, accounted for unique variance in the association between income and self-rated health. We conducted a secondary data analysis of a community sample of 184 adults over the age of 50 in a large, Mid-western city. We tested a mediation model, entering income as a predictor of self-rated health with frequency of physical activity, feelings of being disconnectedness, and ability to find information about needed services as hypothesized mediators, controlling for the effects of race, education, and gender. Access to information about needed services, ab = .08, 95% CI [03, .15] accounted for unique variance in self-rated health scores beyond the direct effect of income, c’ = .38, p < .0001, 95% CI [.22, .53]. These findings suggest that higher perceived access to information about needed services may partially explain the association between income and self-rated health. Future researchers should track these variables across time to establish causal associations between access to information and health. Additionally, further study of perceived access to information and objective measures of access to information is warranted, as the role of health literacy in this study is unclear.

